# Variations in postoperative electrolyte concentrations and influential factors in hip arthroscopy

**DOI:** 10.1186/s12891-022-05451-1

**Published:** 2022-05-19

**Authors:** Guanying Gao, Chang Zhou, Yingfang Ao, Jianquan Wang, Yan Xu

**Affiliations:** Institute of Sports Medicine, Beijing Key Laboratory of Sports Injuries, Peking University Third Hospital, Haidian District, 49 North Garden Road, Beijing, 100191 China

**Keywords:** Hip arthroscopy, Perfusion volume, Electrolyte, Complications

## Abstract

**Background:**

Different from arthroscopy in other joints, more perfusion is required for a better access to perform surgical procedures in hip arthroscopic operations. The significant fluid perfused may lead to complications of partial tissue injury and electrolyte imbalance. However, there were few studies on the change of serum electrolyte after hip arthroscopy and the influential factors were still unknown.

**Methods:**

We evaluated consecutive patients who underwent hip arthroscopy in our hospital between October 2021 and February 2022. Age, sex, and BMI matched patients who underwent arthroscopic anterior cruciate ligament (ACL) reconstruction at the same time were also included as the control group. Preoperative and postoperative serum electrolyte of sodium (Na +), potassium (K +), chloride (Cl-), magnesium (Mg2 +), and carbon dioxide capacity (CO2CP) were analyzed. The correlations between influential factors like perfusion volume, operating time, BMI and hip circumference, and changes in serum electrolyte were also analyzed.

**Results:**

A total of 79 patients were involved in this study, including 49 patients who underwent hip arthroscopy and 30 patients who underwent knee arthroscopy. For hip arthroscopy, decrease of potassium levels was observed in 40.8% of the patients, and postoperative hypokalemia was found in 10.2% patients. There were significant variations in postoperative sodium, magnesium, chloride and carbon dioxide capacity in hip arthroscopy (*p* < 0.05). No correlations were found between each of the electrolyte concentrations and influential factors like perfusion volume, operating time, BMI, sex and hip circumference. The significant variations were found in chloride and carbon dioxide capacity in knee arthroscopy (*p* < 0.05).

**Conclusions:**

Hip arthroscopy would significantly influence postoperative serum electrolyte, and hypokalemia could be a postoperative complication. The correlation between serum electrolyte and fluid perfusion volume is remained to be investigated. We therefor advocate that performing a systematic care of preoperative and postoperative serum electrolyte analysis as perioperative management is necessary.

## Background

Over the past two decades, hip arthroscopy has developed rapidly and has been shown to significantly decrease pain and improve hip function [1–6]. Portal standardization, adequate fluid management, traction time reduction, and improved positioning of the patient on the operating table have reduced the incidence of main complications in the first postoperative year to 1.7% [7, 8]. The major complications include heterotopic ossification, bursitis, proximal femur fracture, intra-abdominal fluid extravasations, postoperative dislocations, temporary neuropraxia, and deep vein thromboses [8–10].

As an important part of arthroscopic procedure, fluid is pressurized to add visualization and dilate the joint or labral. Previous study proved that the correlation of fluid gain and perfusion during the procedure was statistically significant, and the absorption of fluid may lead to pathological outcome [11]. Different from arthroscopy in other joints, more perfusion is required for a better access to perform surgical procedures in hip arthroscopic operations. Hip arthroscopy is capable of larger perfusion volume for the larger space of hip joints [12–14]. The significant fluid perfused may lead to complications of partial tissue injury and electrolyte imbalance [11, 14–16]. Several previous studies reported the electrolyte variations after arthroscopic procedure of shoulder, knee and hip, and the influence of perfusion during the procedure to serum electrolyte is still lack of clinical evidence [11, 14–16]. However, there were few studies on the change of serum electrolyte after hip arthroscopy and the influential factors were still unknown.

The purpose of this study was to investigate the changes in postoperative electrolyte levels after hip arthroscopy and evaluate correlations between electrolyte variations and perfusion volume, operating time and other influential factors in a relative large sample size, using knee arthroscopy as the control group to confirm the particular significance of hip arthroscopy. We hypothesized that the massive absorption of fluid perfused during arthroscopic procedures would lead to significant postoperative electrolyte changes, and correlations were expected to be found between electrolyte changes and influential factors like perfusion volume and operating time.

## Methods

### Patients

We evaluated consecutive patients who underwent hip arthroscopy in our hospital between October 2021 and February 2022 prospectively. Age, sex, and BMI matched patients who underwent arthroscopic anterior cruciate ligament (ACL) reconstruction at the same time were also included as the control group. The inclusion criteria were as follows: (1) patients who underwent hip arthroscopy for femoroacetabular impingement (FAI) in our hospital; (2) age, sex, and BMI matched patients who underwent arthroscopic ACL reconstruction; and (3) had preoperative and postoperative serum electrolyte concentrations. Patients who had previous hip surgeries were excluded from the study. Blood samples were obtained from each patient just before surgery and postoperatively. All participants signed informed consent. The study was approved by the Ethics Committee of the Third Hospital of Peking University. All methods were performed in accordance with the guidelines and regulations of the Ethics Committee of the Third Hospital of Peking University.

### Surgical Procedure

One surgeon with more than 15 years of experience performed standard hip arthroscopy for all patients. All surgeries were performed using a standard supine approach as described by Gao et al. [17] In brief, the interportal capsulotomy technique was used to access the hip joint using the anterolateral and midanterior portals. Then a detailed inspection of the central compartment was performed to assess the acetabular rim, acetabular labrum, articular cartilage, and ligamentum teres. Labral repair or labral debridement was performed according to the nature of injury. If a cam bump in the head-neck junction or acetabular overcoverage was identified, femoral osteoplasty or acetabuloplasty was performed. Capsular closure was routinely done at the end of surgery. 2,000 mL of 0.9% sodium chloride at height of 2 m was used to provide perfusion pressure and the pressure pump was not used. The volume of perfusion during the surgical procedures under hip arthroscopy was recorded. All patients received 1,000 mL of 0.9% sodium chloride solution intravenously during the surgery. No prophylactic antibiotics were administered. Meanwhile, arthroscopic single-bundle ACL reconstruction was performed by the same surgeon as previously described [18]. In brief, tourniquet was routinely used during the surgery. The graft was placed at the center of the whole ACL footprint according to the osseous landmarks and arthroscopic findings of the ACL footprint with the knee in 120° of flexion. The femoral tunnel was drilled through an anteromedial portal. The tibial tunnel was made using a tibial guide through the center of the ACL tibial footprint. The semitendinosus and gracilis tendons were harvested, doubled (7–9 mm), and inserted through the tibial tunnel and into the femur. The graft was fixed using an EndoButton (Smith & Nephew) on the femur and using 1 bioabsorbable interference screw (Smith & Nephew) and 1 staple on the tibia. Every patient received serum electrolyte test as soon as the surgical procedure finished to have the postoperative electrolyte recorded.

### Statistics

The nonparametric Wilcoxon signed rank test was used to compare the preoperative and postoperative levels of sodium (Na^+^), potassium (K^+^), chloride (Cl^−^), magnesium (Mg^2+^), and carbon dioxide capacity (CO_2_CP). The electrolyte variations of different sexes were also compared by Wilcoxon signed rank test. The correlations between perfusion volume, operating time, BMI, hip circumference and changes in electrolyte concentrations were tested by nonparametric Spearman Rank Correlation Coefficient (r). All statistics analysis was done by IBS SPSS Statistics software, version 27. The *p*-values < 0.05 were considered statistically significant.

## Results

As shown in Table 1, this study enrolled 79 patients, including 49 patients (22 men and 27 women; median age, 33 years, range, 15 to 58 years) who underwent hip arthroscopy and 30 patients (14 men and 16 women; median age, 32 years, range, 16 to 53 years) who underwent knee arthroscopy. In 49 patients who underwent hip arthroscopy, all (100%) patients were diagnosed with FAI and 47 (95.9%) patients were diagnosed with labral tear (Table 1).

**Table 1:** Demography of patients (*n* = 85)

Parameter	Data
Patients who underwent hip arthroscopy	
Number	49
Age, y, median (range)	33 (15–58)
Sex	
Male	22 (44.9%)
Female	27 (55.1%)
BMI, kg/m^2^, mean (range)	23.2 (17.1–33.2)
Hip circumference, cm, mean (range)	100.5 (82–138)
Diagnosis	
Femoroacetabular impingement	49 (100%)
Labral tear	47 (95.9%)
Patients who underwent knee arthroscopy	
Number	30
Age, y, median (range)	32 (16–53)
Sex	
Male	14 (46.7%)
Female	16 (53.3%)
BMI, kg/m^2^, mean (range)	23.6 (17.6–33.8)

Unless otherwise specified, data are numbers of patients, with percentages in parentheses

The median perfusion volume of isotonic saline in hip arthroscopy was 2000 ml (range, 200 to 5000 ml). The median operating time was 131 min (range, 93 to 282 min). The median concentrations of electrolyte concentrations were also recorded (Table 2). Preoperative and postoperative electrolyte concentrations in patients who underwent ACL reconstrution as control group was shown in Table 3. None of the patients developed postoperative complications.

**Table 2:** Preoperative and postoperative electrolyte values of hip arthroscopy

	Pre-op		Post-op		MeanΔ	*P* Value
	Median	Range	Median	Range		
K^+^	3.91	3.12–4.87	3.89	2.00–4.62	-0.020	.747
Na^+^	142.75	137.20–145.90	140.22	133.50–146.40	-2.531	.000
Cl^−^	103.91	98.90–108.90	107.00	102.00–125.90	+ 3.091	.000
Mg^2+^	0.88	0.78–1.03	0.80	0.41–0.95	-0.080	.000
CO_2_CP	26.20	21.30–31.50	23.60	14.30–28.00	-2.602	.000

*Abbreviations*: *meanΔ* difference between preoperative value and postoperative value, *Na*^+^ sodium, *K*^+^ potassium, *Cl*^*−*^ chloride, *Mg*^*2*+^ magnesium, *CO*_*2*_*CP* carbon dioxide capacity

**Table 3:** Preoperative and postoperative electrolyte values of knee arthroscopy

	Pre-op		Post-op		MeanΔ	*P* Value
	Median	Range	Median	Range		
K^+^	4.09	3.64–4.58	3.91	3.30–4.27	-0.186	.114
Na^+^	142.85	137.30–145.60	141.68	137.60–144.80	-1.170	.203
Cl^−^	103.90	100.40–108.30	106.88	103.60–109.70	+ 2.980	.005
Mg^2+^	0.90	0.76–1.01	0.89	0.82–0.98	-0.009	.574
CO_2_CP	28.10	24.30–30.90	25.96	22.60–29.00	-2.140	.013

*Abbreviations*: *meanΔ* difference between preoperative value and postoperative value, *Na*^+^ sodium, *K*^+^ potassium, *Cl*^*−*^ chloride, *Mg*^*2*+^ magnesium, *CO*_*2*_*CP* carbon dioxide capacity

Among all preoperative and postoperative electrolyte concentrations tested, decrease of potassium levels was observed in 40.8% of the patients (*n* = 20), and postoperative hypokalemia was found in 10.2% patients (*n* = 5). However, the variation was not significant with a mean decrease of 0.019 ± 0.52 mmol/L (*P* = 0.747). The significant differences were observed in the variations of all other electrolyte concentrations. The decrease in sodium levels was 1.626 ± 2.81 mmol/L (*p* = 7.53E-05), magnesium levels 0.078 ± 0.08 mmol/L (*p* = 2.21E-09), and carbon dioxide capacity 2.604 ± 3.23 mmol/L (*p* = 1.87E-07). Meanwhile, the concentration of chloride level increased by 3.067 ± 3.47 mmol/L (*p* = 2.21E-08). No significantly difference was found in the electrolyte variations of different sexes (*p* = 0.543). No significant correlations were found between perfusion volume and each of the electrolyte concentrations (Na^+^, *r* = 0.066, *p* = 0.685; K^+^, *r* = -0.232, *p* = 0.08; Cl^−^, *r* = 0.008, *p* = 0.986; Mg^2+^, *r* = -0.064, *p* = 0.697; CO_2_CP, *r* = 0.047, *p* = 0.731). There were also no significant correlations between operation time (Na^+^, *r* = 0.076, *p* = 0.785; K^+^, *r* = 0.084, *p* = 0.568; Cl^−^, *r* = 0.018, *p* = 0.886; Mg^2+^, *r* = 0.009, *p* = 0.976; CO_2_CP, *r* = 0.027, *p* = 0.831), BMI (Na^+^, *r* = 0.184, *p* = 0.178; K^+^, *r* = -0.190, *p* = 0.24; Cl^−^, *r* = 0.022, *p* = 0.893; Mg^2+^, *r* = -0.036, p = 0.825; CO_2_CP, *r* = 0.059, *p* = 0.670), hip circumference (Na^+^, *r* = -0.184, *p* = 0.410; K^+^, *r* = 0.228, *p* = 0.124; Cl^−^, *r* = -0.110, *p* = 0.542; Mg^2+^, *r* = -0.089, *p* = 0.623; CO_2_CP, *r* = 0.248, *p* = 0.092) and electrolyte variations.

In the control group, 30 patients were enrolled into analysis. The decrease of potassium was observed in 12 (40%) patients, and 1 (3.3%) patient suffered from postoperative hypokalemia. The concentration variations of potassium (*p* = 0.114), sodium (*p* = 0.203), and magnesium (*p* = 0.574) were not significant. The significant variations were only found in chloride (*p* = 0.005) and carbon dioxide capacity (*p* = 0.013). There was no significant difference in preoperative serum electrolyte between patients who underwent hip arthroscopy and patients who underwent knee arthroscopy (*p* < 0.05). Patients who underwent hip arthroscopy had a higher change of Na^+^ (*p* = 0.0498) and Mg^2+^ (p = 7.55E-05) comparing with patients who underwent knee arthroscopy. There was no significant difference in change of K^+^ (*p* = 0.0498), Cl^−^, and CO_2_CP.

## Discussion

In this study, we evaluated the change of electrolytes before and after hip arthroscopy, significant differences were found in Na + , Cl^−^, Mg^2+^ and carbon dioxide capacity (*p* < 0.05). Meanwhile, 40.8% of the patients who underwent hip arthroscopy suffered a decrease of postoperative potassium concentrations, and 5 of them went through postoperative hypokalemia. However, no correlation was confirmed between electrolyte variations and influential factors like perfusion volume and operating time, and the variation of postoperative K^+^ was not statistically significant.

Isotonic saline perfusion is an essential procedure in arthroscopic surgeries. The effect in hip arthroscopy is consumed to be more significant for its larger perfusion volume capable. The absorption of fluid during arthroscopic procedure would lead to massive hormonal disorders and the variation of electrolyte concentration is of significant pathological value. Patients who go through hypokalemia would suffer from the decrease of neuromuscular excitability, disorder of heart and renal functions, and imbalance of acid–base levels [19]. Other electrolytes imbalances, however, would also lead to postoperative complications. Patients who go through hyponatremia would suffer from general edema for the decrease of serum osmotic pressure, and relevant complications including encephaledema, digestive abnormality and neuromuscular complications would occur [20]. Besides, hyperchloremia is closely related to metabolic acidosis, and may cause digestive complications [21]. Hypomagnesemia may lead to neuromuscular and cardiovascular complications and pathological calcium metabolism for its depression of nerve excitability [22]. The decrease of carbon dioxide capacity would indicate complications of respiratory alkalosis, leading to relative respiratory and acid–base disturbance [23].

In open surgeries, potassium and other electrolyte levels are strictly managed in perioperative period [24–27]. The electrolyte management is also essential in the field of arthroscopic operations. The influence of perfusion in shoulder arthroscopy has been investigated, and significant influence has been certificated between saline perfusion and postoperative volume load [11]. The patients’ hemodynamics psychology and electrolyte concentrations are advocated to require specific attention [11, 14, 28, 29]. Smith, et al. [11] investigated the fluid and electrolyte balance after arthroscopy procedure of the shoulder. The study indicated that there is significant absorption of fluid perfused during the arthroscopic procedure. However, only few previous studies reported the electrolyte variations after arthroscopic procedure of knee and hip, and the influence of perfusion during the procedure to serum electrolyte is still lack of clinical evidence [11, 14–16]. As far as we know, only 2 studies concentrated on serum electrolyte after hip arthroscopy, and they were limited to their relative small sample size. Bernardo et al. [7] compared the differences of postoperative hemodynamic parameters, including electrolyte concentrations and other early complication incidences in bilateral and unilateral hip arthroscopy. Significant differences of sodium levels were found in both bilateral and unilateral hip arthroscopies. Besides, the increase of potassium was found in unilateral hip arthroscopy. Verhelet et al. [12] addressed the variation of serum electrolyte levels and renal function after hip arthroscopy, and he tried to investigate the impact of operating time and perfusion volume on electrolyte levels. The significant variation was only found in postoperative sodium levels, and no correlations were reported between perfusion volume, operating time and electrolyte concentrations. However, only 10 patients were enrolled into the study, and the postoperative electrolyte variations were tested 2 days after the operation, which gave patients adequate time to coordinate psychologically. Our study reported a different result from the results reported by Verhelst et al. [12] and significant differences were found in postoperative sodium, chloride, magnesium, and carbon dioxide capacity levels, and 5 patients underwent hypokalemia and required specific postoperative care in our study. The result is deduced to be attributed to the bigger sample size, different solution perfused, and different surgical procedures.

According to our data, it seems that the arthroscopic procedure in hips would cause significant postoperative variations in serum electrolyte concentrations. There is significant decrease in sodium, magnesium and carbon dioxide capacity levels, and significant increase in chloride level. The variations were consistent in the patients who went through knee arthroscopy, but only the variations in chloride and carbon dioxide capacity were significant, which indicated that hip arthroscopy would exert more significant influence on general serum electrolyte balance. The fluid perfusion absorbed is consumed to be responsible to the general decrease of serum electrolyte concentrations, which may increase the risk of complications of hyponatremia and other hydro-salinity imbalance. The increase of chloride is deduced to be related to the solute of perfusion and psychological negative feedback. However, no significant correlations were found between electrolyte variations and perfusion volume, operating time, sex, BMI and hip circumference, which was different from our anticipation. Nevertheless, the fluid absorbed would psychologically influence general hydro-salinity balance. It is safe to advocate a more delicate management of fluid perfusion, and monitor the postoperative serum electrolyte to prevent from relevant complications.

In this study, although the decrease of potassium was not significant, 40.8% of patients went through the decrease of potassium concentrations, and 5 of them suffered from postoperative hypokalemia, which is anticipated to increase the risk of severe complications. In knee arthroscopy, the decrease of potassium was observed in 12 (40%) patients, and 1 (3.3%) patient suffered from postoperative hypokalemia. The percent of patients who underwent hypokalemia was much higher in hip arthroscopy. So we thought the postoperative potassium levels should be evaluated to prevent from serious electrolyte complications.

### Limitations

This study had several limitations. First, although the sample size was enough to detect differences in change of electrolytes before and after hip arthroscopy, this sample can be considered small to detect differences in other influential factors. Second, the perfusion volume of knee arthroscopy in ACL reconstruction was not calculated. However, the perfusion volume of knee arthroscopy was relatively small and we thought it may have little influence on the results.

## Conclusions

Hip arthroscopy would significantly influence postoperative serum electrolyte, and hypokalemia could be a postoperative complication. The correlation between serum electrolyte and fluid perfusion volume is remained to be investigated. We therefor advocate that performing a systematic care of preoperative and postoperative serum electrolyte analysis as perioperative management is necessary.

## Data Availability

All relevant data supporting the conclusions are included within the article and tables. The datasets used and/or analysed during the current study available from the corresponding author on reasonable request.

## References

[1] Gohal C, Shamshoon S, Memon M, Kay J, Simunovic N, Randelli F, Ayeni OR (2019). Health-Related Quality of Life After Hip Arthroscopy for Femoroacetabular Impingement: A Systematic Review and Meta-analysis. Sports Health.

[2] Minkara AA, Westermann RW, Rosneck J, Lynch TS (2019). Systematic Review and Meta-analysis of Outcomes After Hip Arthroscopy in Femoroacetabular Impingement. Am J Sports Med.

[3] Menge TJ, Briggs KK, Dornan GJ, McNamara SC, Philippon MJ (2017). Survivorship and Outcomes 10 Years Following Hip Arthroscopy for Femoroacetabular Impingement: Labral Debridement Compared with Labral Repair. J Bone Joint Surg Am.

[4] Menge TJ, Briggs KK, Rahl MD, Philippon MJ (2021). Hip Arthroscopy for Femoroacetabular Impingement in Adolescents: 10-Year Patient-Reported Outcomes. Am J Sports Med.

[5] Wolfson TS, Ryan MK, Begly JP, Youm T (2019). Outcome Trends After Hip Arthroscopy for Femoroacetabular Impingement: When Do Patients Improve?. Arthroscopy.

[6] Nho SJ, Beck EC, Nwachukwu BU, Cvetanovich GL, Neal WH, Harris JD, Weber AE, Mather RC (2019). Survivorship and Outcome of Hip Arthroscopy for Femoroacetabular Impingement Syndrome Performed With Modern Surgical Techniques. Am J Sports Med.

[7] Aguilera-Bohorquez B, Pachon M, Sanchez M, Ramos-Cardozo O, Cantor E (2019). Metabolic and Hemodynamic Results and Early Complications in Simultaneous Bilateral versus Unilateral Hip Arthroscopy. Clin Orthop Surg.

[8] Truntzer JN, Hoppe DJ, Shapiro LM, Abrams GD, Safran M (2017). Complication Rates for Hip Arthroscopy Are Underestimated: A Population-Based Study. Arthroscopy.

[9] Kowalczuk M, Bhandari M, Farrokhyar F, Wong I, Chahal M, Neely S, Gandhi R, Ayeni OR (2013). Complications following hip arthroscopy: a systematic review and meta-analysis. Knee Surg Sports Traumatol Arthrosc.

[10] Harris JD, McCormick FM, Abrams GD, Gupta AK, Ellis TJ, Bach BR, Bush-Joseph CA, Nho SJ (2013). Complications and Reoperations During and After Hip Arthroscopy: A Systematic Review of 92 Studies and More Than 6,000 Patients. Arthroscopy-the J Arthroscopic Related Surg.

[11] Smith CD, Shah MM (2008). Fluid gain during routine shoulder arthroscopy. J Shoulder Elbow Surg.

[12] Verhelst L, De Schepper J, Sergeant G, Liekens K, Delport H (2009). Variations in serum electrolyte concentrations and renal function after therapeutic hip arthroscopy: a pilot study. Arthroscopy.

[13] Haupt U, Völkle D, Waldherr C, Beck M (2008). Intra- and Retroperitoneal Irrigation Liquid After Arthroscopy of the Hip Joint. Arthroscopy.

[14] Lo IKY, Burkhart SS (2005). Immediate Postoperative Fluid Retention and Weight Gain After Shoulder Arthroscopy. Arthroscopy.

[15] Christ F, Moser C, Peter K, Messmer K (1998). Changes in fluid filtration capacity and blood flow Tourniquet used for knee operations. Anaesthesist.

[16] Saeed NTM, Azam M (2009). Rhabdomyolysis secondary to interaction between atorvastatin and fusidic acid. BMJ case rep.

[17] Gao G, Zhang X, Xu Y, Wang J (2019). Clinical outcomes and causes of arthroscopic hip revision surgery. Sci Rep.

[18] Lin L, Wang H, Wang Y, Wang J, Liu Y, Yu J (2022). Double-Bundle Versus Single-Bundle Anterior Cruciate Ligament Reconstruction in Patients With Significant Passive Anterior Tibial Subluxation. Am J Sports Med.

[19] Ponikowski P, Voors AA, Anker SD, Bueno H, Cleland JGF, Coats AJS, Falk V, Gonzalez-Juanatey JR, Harjola VP, Jankowska EA (2016). 2016 ESC Guidelines for the diagnosis and treatment of acute and chronic heart failure. Eur Heart J.

[20] Adrogué HJ, Madias NE (2000). Hyponatremia.

[21] Bullivant EM, Wilcox CS, Welch WJ (1989). Intrarenal vasoconstriction during hyperchloremia: role of thromboxane. Am J Physiol.

[22] Agus ZS (1999). Hypomagnesemia. J Am Soc Nephrol.

[23] Adrogué HJ, Madias NE (2010). Secondary responses to altered acid-base status: the rules of engagement. J Am Soc Nephrol.

[24] Ali SM, Shaikh N, Shahid F, Shah A, Zafar HB (2020). Hypokalemia Leading to Postoperative Critical Arrhythmias: Case Reports and Literature Review. Cureus.

[25] Lai X, Zhang H, Wang H (2021). Multivariate Analysis of Hypokalemia in Patients With Primary Hypertension After Total Knee Arthroplasty. Chin J Minimally Invasive Surg.

[26] Swearingen AJ, Kahramangil B, Monteiro R, Krishnamurthy V, Jin J, Shin J, Siperstein A, Berber E (2018). Analysis of postoperative biochemical values and clinical outcomes after adrenalectomy for primary aldosteronism. Surgery.

[27] Xu B, Xu WX, Lao YJ, Ding WG, Lu D, Sheng HF (2019). Multimodal Nutritional Management in Primary Lumbar Spine Surgery A Randomized Controlled Trial. Spine.

[28] Ogilvie-Harris DJ, Boynton E (1990). Arthroscopic acromioplasty: Extravasation of fluid into the deltoid muscle. Arthroscopy.

[29] Sperber A, Wredmark T (1999). Intramuscular pressure and fluid absorption during arthroscopic acromioplasty. J Shoulder Elbow Surg.

